# Accurate and fully automated diameter measurements of Circle of Willis arteries on MRA imaging

**DOI:** 10.1177/0271678X251338972

**Published:** 2025-05-05

**Authors:** Julia Huck, Davy Vanderweyen, Tatjana Rundek, Mitchell SV Elkind, Jose Gutierrez, Maxime Descoteaux, Kevin Whittingstall

**Affiliations:** 1 Department of Nuclear Medicine and Radiobiology, Université de Sherbrooke, Sherbrooke, CA, USA; 2 Department of Diagnostic Radiology, Université de Sherbrooke, Sherbrooke, CA, USA; 3 Evelyn F. McKnight Brain Institute and Department of Neurology, Miller School of Medicine, University of Miami, Miami, USA; 4 Department of Neurology, Vagelos College of Physicians and Surgeons, Columbia University, New York, USA; 5 Department of Epidemiology, Mailman School of Public Health, Columbia University, New York, USA; 6 Department of Computer Science, Université de Sherbrooke, Sherbrooke, CA, USA

**Keywords:** Automated vascular segmentation, cerebral artery validation, cerebral diameter estimation, cerebral diameter validation, Circle of Willis

## Abstract

The Circle of Willis (CW), visualized via Magnetic Resonance Angiography (MRA), is crucial for assessing cerebral circulation. Accurate artery identification is essential not only for detecting stenosis and pathological changes but also for understanding vascular adaptations in healthy aging. Manual CW assessment is time-consuming, necessitating automated alternatives. This study evaluates intracranial artery diameter estimations from the Express IntraCranial Arteries Breakdown (eICAB) toolbox against manual measurements. eICAB was tested on 631 participants from the Northern Manhattan Study (NOMAS) with 1.5T MRA images (0.293 × 0.293 × 1 mm resolution). We analyzed eICAB’s detection and diameter estimation accuracy of the Internal Carotid (ICA), Basilar (BA), Anterior Cerebral (ACA), Middle Cerebral (MCA), Posterior Cerebral (PCA), and Posterior Communicating (PCom). eICAB showed over 95% accuracy in detecting major arteries except for PCA and PCom (∼80%). Diameter discrepancies were generally ≤0.5 mm, with ICA and BA reaching 1 mm. Spearman correlation (p ≪ 0.05) confirmed strong agreement between automated and manual measurements. Resampling at 0.2083 mm improved precision. eICAB accurately identifies CW arteries and estimates diameters, demonstrating strong clinical and research potential.

## Introduction

The human brain is in constant need of oxygen, glucose and other nutrients delivered by the arterial blood, which is supplied by large extracranial arteries: the right and left internal carotid and the basal artery, which is supplied by the vertebral arteries. These arteries are connected at the base of the skull by a large anastomotic polygon, commonly referred to as the Circle of Willis (CW).^1^ Time-of-flight (TOF) magnetic resonance angiography (MRA) and computed tomography angiography (CTA) are the most widely used modalities for visualizing human CW in basic and clinical research.^2^ Accurate identification and measurement of the arteries within the CW are crucial for diagnosing and treating various cerebrovascular diseases, such as anatomical variations, pathological narrowing (stenoses), and other pathologies that can impact the cerebral circulation.^3^ Stenoses of the intracranial arteries restrict the passage of oxygen-rich blood flow, significantly increasing the risk of stroke,^4–6^ dementia^7,8^ and death.^4^ Traditionally, diameter measurements are made manually by medical specialists, a process that is both time-consuming and prone to inter- and intra-rater variability.^3^ Automated measures of CW diameters could potentially lead to better clinical outcomes by facilitating early and precise diagnosis, guiding therapeutic decisions, and monitoring disease progression. Moreover, TOF imaging is increasingly included in large cohort studies such as Open Access Series of Imaging Studies (OASIS),^9^ yet without automated tools, analyzing large datasets is impractical due to the labor-intensive nature of manual annotation. With the rising use of advanced imaging and the likelihood of incidental findings, automated methods hold significant potential for streamlining detection, saving time and labor, and enabling consistent analysis across research and clinical settings.

In this study, we conducted a comprehensive evaluation of a recently developed method Express IntraCranial Arteries Breakdown (eICAB) designed to automatically identify and measure the diameters of the arteries within the CW^10^ on MRA imaging. The primary objective of this study is to assess the accuracy of the algorithm in identifying these arteries and determining their diameters, compared to the manual measurements performed by medical experts. Our goal is to validate our algorithm using the Northern Manhattan Study (NOMAS) dataset, which is one of the largest and most diverse datasets featuring expert manual measurements of arteries in the Circle of Willis, spanning a wide range of image qualities and participant demographics.

## Materials and methods

### Participants

Participants were selected from NOMAS, a multi-ethnic, population-based prospective cohort that includes a total of 3,298 individuals aged 40 years and older, residing in Northern Manhattan. Methods of the NOMAS study have been previously described.^11^ Of the total cohort, 1,290 participants underwent MRI imaging, and from this group, 631 individuals without pathological narrowing (stenosis) identified by a vascular neurologist (JG) were chosen for this analysis. These participants had an average age of 69.7 ± 8.4 years (mean ± SD, 364 females). Ethical approval for the study was secured from the institutional review boards of Columbia University and the University of Miami. All subjects signed informed consent. The study was conducted in accordance with the ethical principles outlined in both the Declaration of Helsinki and the Belmont Report.

### Scanning Protocol

Acquisitions were completed on a 1.5 T Philips Gyroscan Intera MR system at Columbia University. Magnetic resonance angiography (MRA) images were acquired with a resolution of 0.293 × 0.293 × 1 mm; repetition time (TR) = 20 ms; echo time (TE) = 2.7 ms; matrix = 512 × 512 × 97; FOV of 15 cm; 1 mm effective slice thickness; Flip Angle = 25°; and an acquisition time (TA) = 12:11 min. The scanning protocol used a single slab positioned from the carotid artery to the top of the Circle of Willis to ensure full coverage of the intracranial arteries.

### Measurements of the arteries

**Figure 1.**
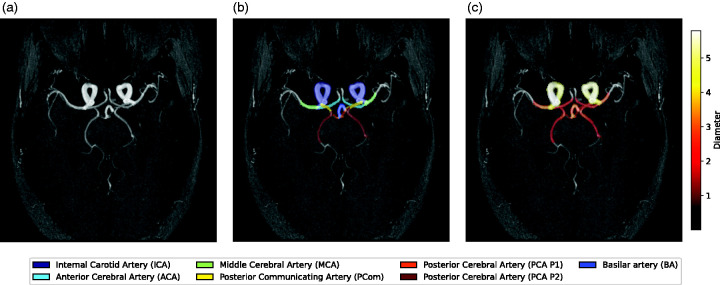
Example of one of the participants. (a) Maximum intensity projection of the TOF image in axial projection; (b) TOF image overlayed with the eICAB segmentations (for representation purposes, the P2 segment of the PCA is also depicted) and (c) TOF image overlayed with the diameter estimations from eICAB.

We used eICAB to analyze the right and left intracranial internal carotid arteries (ICAs), M1 segment of middle cerebral arteries (MCAs), A1 segment of anterior cerebral arteries (ACAs), P1 segments of posterior cerebral arteries (PCAs), and Posterior communicating arteries (PComs), and the basilar artery (BA) in MRA images^10^ (Figure 1(a) and (b)). The anterior communicating artery (ACom) was not included in this study. The eICAB algorithm is a fully automated tool using convolutional neural network (CNN) models. The CNN models of eICAB were trained on a heterogeneous dataset of 116 MRA (31 were acquired locally (CR-CHUS), 20 were taken from the MIDAS^12^ and 65 from the OASIS-3 database^9^). All datasets were acquired on 3T MRI machines with various resolutions: data from the CR-CHUS had a resolution of 0.625 × 0.625 × 0.65 mm^3^, and 0.51 × 0.51 × 0.80 mm^3^ and 0.30 × 0.30 × 0.60 mm^3^ for the MIDAS and OASIS dataset, respectively. On these images, fourteen arterial segments of the CW were manually annotated and validated by a clinical expert. Before feeding the MRA images to the CNN, the preprocessing steps of the data included a reorientation of the images into RAS orientation, skull-stripping and a resampling to 0.625 mm^3^. A spherical region of interest (ROI) spanning the CW is extracted. The CNN inference is then applied to this extracted patch, resulting in a segmented CW with up to 14 anatomical labels. Next, voxel-wise diameter estimates are made by calculating local thickness for each blood vessel voxel within the binary segmentation mask by fitting the largest possible sphere. Diameter values are then assigned to each voxel^13^ (Figure 1(c)). Subsequently, the centerlines of the blood vessels are computed, and only the diameter values along these centerlines are considered for the final diameter estimate. The diameter values were averaged for the whole length of the segments, yielding one diameter value per artery. For comparative purposes, the images were first 3D reconstructed using a surface modeling software (LAVA; Leiden University Medical Center, the Netherlands) before manual measurements were performed.^14,15^ The diameters of each artery were also manually measured in the native MRA resolution of 0.293 × 0.293 × 1.000 mm^3^ at a single point along the artery by a vascular neurologist (author JG) using a digital caliper. Cross-sectional views perpendicular to the vessel’s long axis were obtained using LAVA, ensuring precise caliper-based measurements. The specific anatomical locations for these single-point measurements were are follows: the cavernous portion of the ICA; within the proximal 5 mm of the MCA M1, ACA A1, and PCA P1; and the proximal portion of the PComs; and the proximal 5 mm of the BA.^16–19^ The Shapiro-Wilk test showed that the data was not normally distributed. Measurements for the right and left arteries, if applicable, were added as separate measurements. For each artery, three analyses were carried out: (1) To determine accuracy, we computed the difference between manual and eICAB diameter measurements at five isotropic resampled image resolutions using a nearest neighbor interpolation (0.2083, 0.3125, 0.4, 0.5 and 0.625 mm^3^). (2) To test for clinical relevance, we used a Wilcoxon Signed-Rank Test to determine whether these differences were within 0.5 mm, and if not, whether they were at least within 1 mm, which are common values generally used to reflect typical intra-rater variability.^20,21^ A Bonferroni correction was performed to adjust for multiple comparisons. (3) Finally, to determine shared variability, we computed Spearman correlation coefficients between all eICAB and manual measurements (N = 631 measurements per artery). Additionally, the accuracy of the diameter estimates, compared to the ground truth, was assessed in a subset of individuals with at least one fetal PCA and compared to the entire cohort (Table S2). A further comparison between the diameter accuracy in the right and left hemispheres was also conducted (Table S3).

## Results

### Accuracy of artery identification

**Figure 2.**
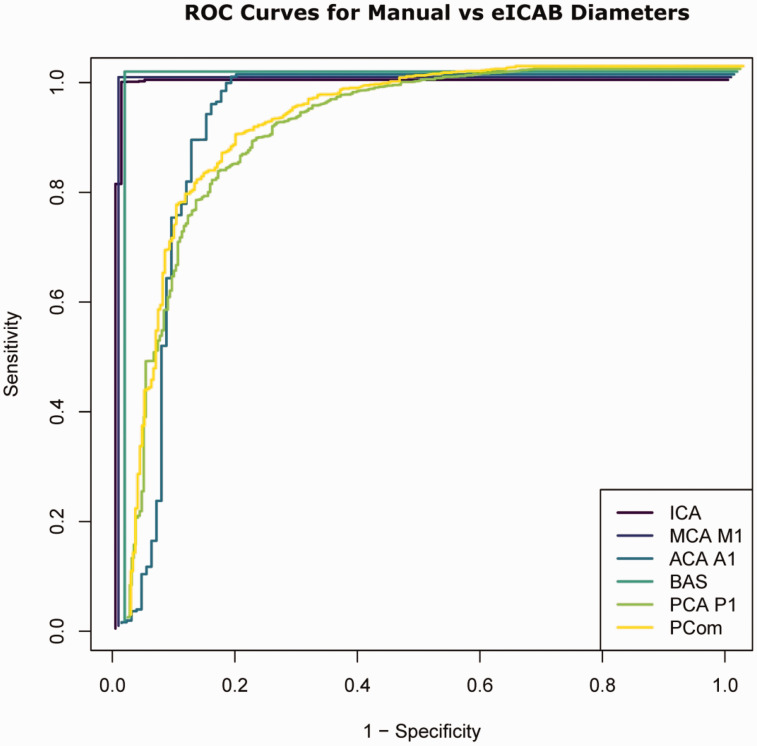
Receiver Operating Characteristic (ROC) curves are presented for each artery at a resampled voxel size of 0.625 mm^3^, illustrating the trade-off between the proportion of correctly classified arteries (sensitivity or True Positive Rate) and the proportion of incorrectly classified arteries (1 - specificity or False Positive Rate).

The performance of eICAB in correctly detecting CW arteries is summarized in Figure 2 and Table 1. When both the eICAB algorithm and the medical expert, considered as the ground truth, provided valid measurements for an artery, it was classified as a true positive. Conversely, if neither method provided a measurement for an artery, it was considered a true negative. Instances where the expert recorded a measurement, but the eICAB algorithm did not were classified as false negatives, while cases in which the eICAB algorithm provided a measurement but the vascular neurologist did not were categorized as false positives.

**Table 1.** Accuracy of artery measurement outcomes based on the agreement between the eICAB algorithm and vascular neurologist assessments. True positives are arteries validated by both methods, true negatives are arteries unmeasured by both, false negatives are arteries measured only by the vascular neurologist, and false positives are arteries measured only by the eICAB algorithm.

Artery	True positive/True negative	False negative	False positive
ICA	99.52%	0.48%	0.00%
ACA A1	94.77%	5.15%	0.08%
MCA M1	99.21%	0.79%	0.00%
PCom	81.22%	17.67%	1.11%
PCA P1	78.37%	20.21%	1.51%
BA	99.84%	0.16%	0.00%

The receiver operating characteristic (ROC) curve in Figure 2 demonstrates a high true positive rate (sensitivity) across all arteries relative to the false positive rate (1 - specificity), indicating strong classification performance for manually measured versus eICAB diameters. eICAB achieved high accuracy in identifying the arteries, with agreement rates exceeding 95% for the ICA, BA, ACA, MCA and ACA. PCA and PCom accuracy were lower, near ∼80% (Table 1). The false negative rate was below 6% for all arteries, except for the PCom and PCA P1 where the false negative rate was ∼20%. The false positive rate was below 2% for all arteries.

### Accuracy of diameter estimation

#### Influence of voxel size

**Figure 3.**
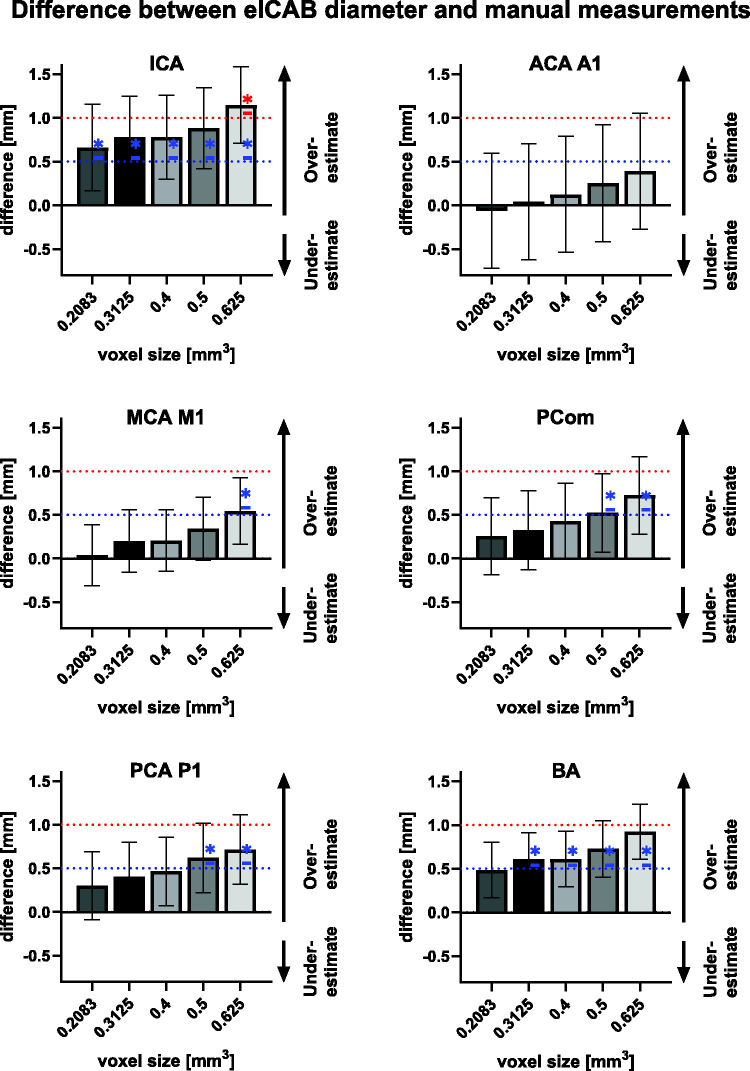
Comparison of artery diameter measurements between the eICAB algorithm and manual assessments across different resampled voxel sizes (voxel sizes: 0.2083, 0.3125, 0.4, 0.5, 0.625 mm). It illustrates the measurement discrepancies for each artery, including the ICA, ACA A1, MCA M1, PCom, PCA P1, and BA. Wilcoxon Signed-Rank Test evaluated the significance of differences being larger than 0.5 mm and 1 mm (blue and red dotted lines, respectively). Significant differences (p < 0.05) are marked with a star (*) for each resampled voxel size, adjusted for multiple comparisons using Bonferroni correction.

Figure 3 illustrates the differences between manually measured diameters and those estimated by the eICAB algorithm at different voxel resolutions. The average diameter difference for all arteries combined were 0.26 ± 0.54 at 0.2083 mm^3^ voxel size, 0.42 ± 0.25 at 0.3125 mm^3^, 0.47 ± 0.26 at 0.4 mm^3^, 0.59 ± 0.22 at 0. 5 mm^3^ and 0.77 ± 0.25 at 0.625 mm^3^. The differences per artery and their relative error are reported in Table S2 in the supplemental material. The mean and standard deviation (std) diameters of the manual and eICAB measurement are listed in Table 2. At each resolution, eICAB diameter estimates were less than 0.5 mm for the ACA (p = 6.52e-103), PCA (p = 4.75e-12) and MCA (p = 2.61e-149). For the ICA and BA, diameter errors were systemic higher but remained globally within 1 mm, except for the ICA at 0.625 mm resolution, where the diameter error reached approximately +1 mm. To assess the clinical relevance of these findings, we examined the proportion of participants whose diameter estimates fell within 0.5 mm and 1 mm of the expert's measurements, as these thresholds are of critical interest and reflect typical intra-rater variability.^20,21^ Across all arteries, approximately 55% of diameter differences were less than 0.5 mm, while 92% were within 1 mm (Table 3). A notable exception was the ICA, whose diameter estimates were slightly higher than the other arteries. As image resolution decreased, the percentage of participants within these thresholds improved across all arteries, with varying extents. For instance, the MCA M1 segment showed only a 7.9% reduction in participants within the ±1 mm range, whereas the ICA and BA showed reductions of 34.5% and 40%, respectively. The potential impact of anatomical variations was assessed by comparing the diameter differences between the ground truth and the eICAB estimates for the full cohort and a subgroup of individuals with at least one fetal PCA, as defined by our expert (Table S2). The average diameter difference between eICAB measurements and the manual segmentations was within 0.1 mm in the subset of individuals with at least one fetal PCA, compared to the entire cohort.

**Table 2.** Mean and std of the measured arteries (ICA, ACA A1, MCA M1, PCom, PCA P1 and BA) across all participants for the expert measurements and the eICAB estimations at different resampled voxel sizes.

Artery	Manual measurements	0.2083	0.3125	0.4	0.5	0.625
ICA	4.26 ± 0.62	4.89 ± 0.52	5.03 ± 0.55	5.03 ± 0.56	5.15 ± 0.74	5.41 ± 0.76
ACA A1	2.12 ± 0.69	2.07 ± 0.35	2.19 ± 0.37	2.28 ± 0.35	2.40 ± 0.39	2.54 ± 0.36
MCA M1	2.71 ± 0.33	2.75 ± 0.34	2.91 ± 0.39	2.92 ± 0.37	3.06 ± 0.41	3.25 ± 0.76
PCom	1.59 ± 0.81	1.94 ± 0.47	2.04 ± 0.47	2.15 ± 0.46	2.25 ± 0.48	2.43 ± 0.50
PCA P1	1.96 ± 0.75	2.39 ± 0.31	2.46 ± 0.45	2.53 ± 0.42	2.67 ± 0.46	2.80 ± 0.43
BA	2.77 ± 0.51	3.20 ± 0.44	3.35 ± 0.50	3.36 ± 0.49	3.48 ± 0.49	3.67 ± 0.50

**Table 3.** Assessment of diameter estimation accuracy for eICAB compared to manual measurements by a vascular neurologist. The table shows the proportion of participants whose estimated vessel diameters were within 0.5 mm and 1 mm of the reference measurements, across various resampled voxel sizes (0.2083, 0.3125, 0.4, 0.5, 0.625).

	0.5 mm					1 mm				
Artery	0.2083	0.3125	0.4	0.5	0.625	0.2083	0.3125	0.4	0.5	0.625
ICA	31.4%	20.5%	20.4%	15.1%	6.83%	76.1%	65.9%	65.8%	56.5%	31.4%
ACA A1	88.9%	73.9%	70.5%	56.8%	42.9%	99.2%	92.8%	91.4%	90.3%	85.5%
MCA M1	92.3%	77.5%	78.5%	64.9%	40.4%	99.7%	98.9%	98.8%	97.7%	91.0%
PCom	75.8%	64.2%	52.8%	44.0%	20.7%	96.7%	94.5%	92.4%	88.0%	78.8%
PCA P1	73.4%	55.8%	49.7%	31.1%	23.7%	97.7%	95.0%	93.4%	86.1%	77.1%
BA	46.0%	29.7%	30.7%	20.9%	9.26%	97.4%	94.6%	93.3%	81.6%	54.3%

#### Correlations between manual measurements and eICAB diameter estimates at 0.2083 mm resolution

**Figure 4.**
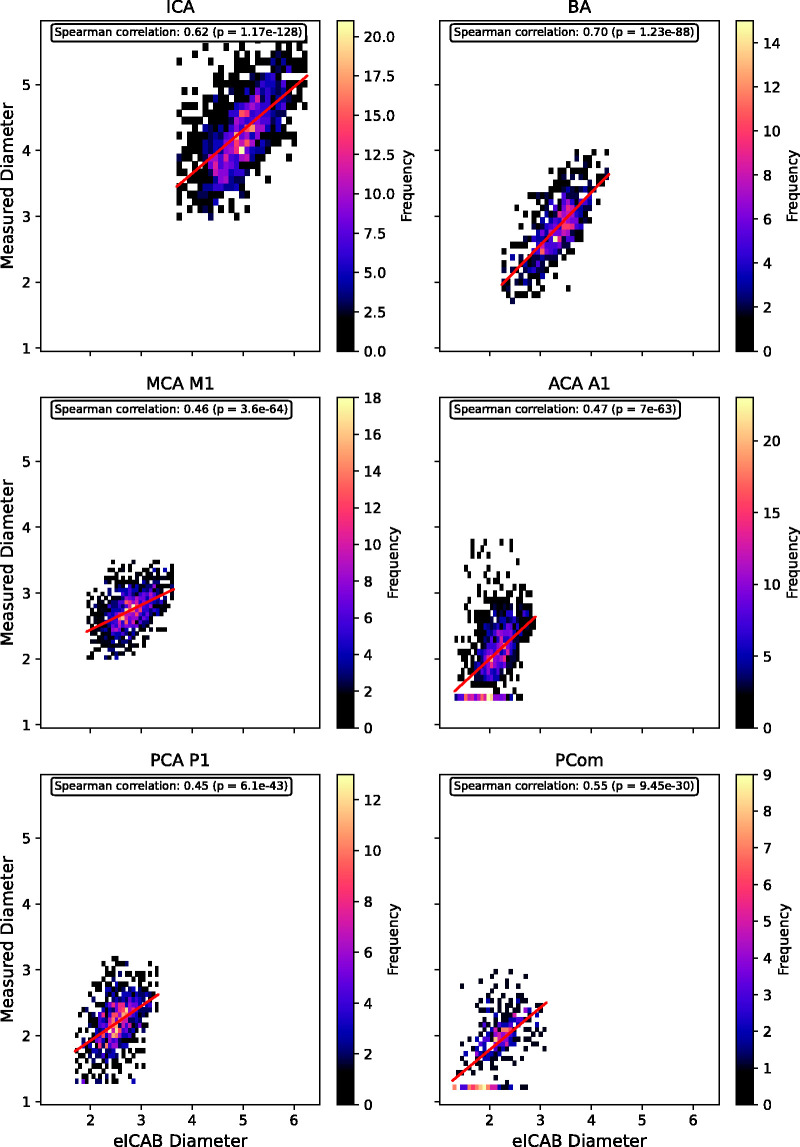
2D histograms plotting manual measurements (y-axis) against eICAB-derived diameters (x-axis) for each artery at a 0.2083 mm resolution, showing the correlation analysis between eICAB diameter estimates and manual measurements. Linear regression lines, depicted in red, illustrate trends and potential overestimations by eICAB. Spearman correlation coefficients indicate the strength and significance of the correlations.

To assess how closely eICAB diameter outputs track human measurements, Spearman’s correlations between the eICAB diameter and the manual measurements are illustrated in Figure 4. The corresponding slope, intercept, R^2^, and RMSE values of the regression lines are provided in Table S1. In each artery, diameter correlations were highly significant (p ≪ 0.05).

## Discussion

In this study, we applied our recently developed method (eICAB) for analyzing the cerebral vasculature on MRA imaging to a large dataset accompanied with ground truth manual diameter measurements. Results show that eICAB diameter estimates align closely with human measurements, thus validating the first (to our knowledge) open-source method for fast and automatic assessment of Circle of Willis arteries in MRA imaging.

### Accuracy of eICAB in detecting arteries within the Circle of Willis

The results indicate that eICAB is reliable in detecting specific arterial segments within the Circle of Willis, achieving a true positive and true negative rate of over 78% across all arteries. The high agreement rates, particularly for major arteries such as the ICA, MCA M1, ACA A1, and BA, where correct detection exceeded 95%, suggest that eICAB's performance in classifying arteries is comparable to that of a medical expert. The system’s low false positive rates further emphasize its precision in accurately identifying arterial structures. Additionally, the resolution did not impact the accuracy of artery detection, highlighting eICAB's robustness across different imaging conditions.

However, the variability in false negative rates points to areas needing improvement, especially for the PCA P1 and PCom arteries. The false negative rates of approximately 20% for these arteries suggest that eICAB may occasionally miss these structures while there was a manual measurement as ground truth, potentially due to anatomical variability, small size, or limitations in image quality. Additionally, only PComs that were connected to both the ICA and PCA were considered. If even one voxel was disconnected from these arteries due to mis-segmentation or intensity fluctuations, the PCom was excluded, contributing to the higher false negative rate.

Dunås et al. reported similar findings with their automatic atlas-based artery identification method (AAIM), where two medical specialists independently assessed the presence of arteries.^22^ Their detection accuracy closely matched the results presented in this paper, differing by only ±2% for most arteries, except for the PCA and PCom. Notably, eICAB outperformed AAIM in detecting the PCom, achieving an accuracy of 81.22% compared to Dunås et al.'s 70%. However, Dunås et al. reported a significantly higher accuracy for the PCA (97% for true positive and true negative), while eICAB’s accuracy for the PCA was 78.37%. This difference likely stems from the fact that Dunås et al. did not differentiate between the various segments of the PCA, such as the P1 and P2 segments, which may have been a factor improving their reported accuracy by considering the artery as a single structure. Additionally, it is important to note that Dunås et al. used 4D flow MRI rather than TOF, which may also contribute to differences in detection accuracy between the two methods.

### Accuracy of the diameter estimation from eICAB compared to manual measurements from a vascular neurologist

To our knowledge, this study is the first to investigate the accuracy of arterial diameter estimations comparing manual measurements, considered as the ground truth, performed by a vascular neurologist against an automated algorithm (eICAB) on a large cohort of participants.

Regarding measurement comparisons and error considerations, the discrepancies between the eICAB algorithm and the manual measurements were within a 0.5 mm error margin for all arteries except the ICA and BA. Given that the intra-rater variability also hovers around 0.5 mm,^20,21^ these discrepancies are within acceptable range, often representing a difference of merely 1–2 voxels at a resampled voxel size of 0.3125 mm. Further, a previous study reported intra-rater differences of 0.5 mm across different raters in the NOMAS dataset,^23^ reinforcing the notion that such variability is inherent even in manual assessments. The results in Table S2 investigating the potential impact of anatomical variations of the CW, indicate that diameter changes are comparable between the full cohort and the fetal PCA subgroup for each artery. This supports the robustness of the eICAB algorithm in detecting arterial diameters, even in the presence of CW variations.

Similarly, a recent systematic review and meta-analysis investigated the variability of intracranial vessel diameters using data from 76 studies.^24^ Diameter measurements from various cerebral arteries were analyzed, and meta-analyses were performed to determine predicted diameter distributions. The findings highlighted substantial variability in vessel diameters across different patient populations and imaging techniques. The review specifically focused on the ICA (overall, C1, and C7 segments), ACA A1, MCA M1, and BA. The mean diameters and standard deviations reported were as follows: 4.74 ± 0.64 mm for the ICA C1 segment (the overall ICA had an average diameter of 4.45 ± 0.72 mm), 1.89 ± 0.34 mm for the ACA A1, 2.55 ± 0.42 mm for the MCA M1, and 2.96 ± 0.52 mm for the BA. The averages of both our manual measurements and eICAB estimates (with resampling to 0.3125 mm) align well with these values, falling within the mean ± standard deviation ranges reported by the systematic review (Table 2). Our findings align with those of Mirza et al., further validating the reliability and consistency of our measurements and demonstrating their agreement with previously published data.

This study further explored the effect of various voxel sizes on the accuracy of arterial diameter estimations via the eICAB algorithm. Findings highlight a notable impact of reconstructed image resolution on the algorithm's accuracy, with a resampled voxel size of 0.2083 mm consistently providing the most precise diameter estimations, as detailed in Table 3.

This observation is critical, given that the eICAB algorithm was initially trained on data with an isotropic resampled voxel size of 0.625 mm.^10^ To ensure consistency with the training data, we chose to evaluate the algorithm at this resolution. Additionally, since accurate diameter estimation requires at least two voxels per vessel, we tested the algorithm at both half (0.3125 mm) and one-third (0.2083 mm) this resolution to improve accuracy by better capturing vessel boundaries and minimizing partial volume effects. A volumetric change from 0.2441 mm³ (for a voxel size of 0.625 mm³) to 0.00904 mm³ (for isotropic voxels of 0.2083 mm³) results in greater detail preservation and improved diameter estimations. However, this comes at the cost of increased computational demand for the algorithm. Moreover, as the algorithm needs at least two voxels in diameter for accurate measurement, this underscores the benefits of higher image resolution, especially for smaller arteries. Insufficient resolution may lead to errors due to partial volume effects, where a single voxel includes multiple tissue types, ultimately skewing the diameter estimation.

The ICA and BA were less accurate compared to the other arteries, reflecting unique challenges in their morphology and algorithmic representation. Specific considerations for the ICA include its tortuous and complex morphology. Notably, the carotid siphon, a segment of the ICA characterized by an S-shaped curve, may be misrepresented as artery segment by eICAB with a very large diameter at greater resolutions^25,26^ due to the phenomenon of “kissing arteries”, where vessels in close proximity appear to merge on MRI images because of voxel resolution limitations. A finer resolution can likely distinguish these loops more accurately, resulting in more precise diameter estimations. However, even at finer resolutions, segments in the carotid siphon remained as apparently touching in some participants, and further work is required to separate these arteries for a more accurate diameter estimation.^27,28^

Further, the impact of variations in the Circle of Willis (CW) was investigated. eICAB showed comparably accurate diameter estimations in individuals with at least one fetal PCA as for the entire cohort (Table S2 in supplemental material). This suggests that the eICAB algorithm is capable of handling anatomical variations typically observed in the CW. These findings highlight the robustness of the eICAB method across a range of vascular configurations, reinforcing its potential for widespread applicability in clinical and research settings where CW variations are common.

### Correlations between manual measurements and eICAB diameter estimates at 0.2083 mm resolution

In the context of our study, Spearman correlation analysis of the eICAB measurements at a 0.2083 mm resolution demonstrated significant correlations with the manual measurements. Moreover, the mean absolute error (MAE) of eICAB is smaller than the reported intra-rater variability of 0.5 mm in this dataset (Table S1).^23^ Our results indicate that eICAB’s diameter estimations are mostly below this 0.5 mm threshold. The slopes, which are consistently less than 1 (Table S1) are in line with our results in Figure 3, showing a systematic overestimation of vessel diameters by the eICAB algorithm compared to manual measurements. Importantly, these findings were consistent regardless of vessel size or variations in the CW, such as having at least one fetal PCA, indicating that the eICAB algorithm's performance is robust and largely independent of these anatomical variations.

## Limitations

One limitation pertains to the criteria used for considering arteries for measurement, particularly for the PCom and the P1 segment of the PCA. The criteria for inclusion of these arteries need refinement to improve the accuracy of their detection rate. These areas require additional methodological development to ensure that the segmentation process is robust and reliable across varying anatomical structures.

The main limitation of this study is the accuracy of the diameter estimation of the ICA and BA. Further improvements are needed to accurately segment the ICA and BA, given their anatomical complexity.

## Conclusion

We demonstrated high accuracy in estimating arterial diameters, closely aligning with expert measurements. While eICAB systematically overestimated diameters, our results showed accuracies within 0.5 mm for the ACA (84% of participants), MCA (81%), PCA (57%), and PCom (60%), and within 1 mm for the ICA (76%) and BA (95%). Accurate diameter estimation is vital for establishing baselines that support the early detection and diagnosis of vascular abnormalities such as stenosis or aneurysms. Although the algorithm does not currently detect these pathologies, achieving precise measurements in healthy participants is a critical step toward this goal. Future developments will focus on incorporating automated detection of vascular pathologies, enabling a comprehensive assessment of cerebrovascular health. By refining our understanding of arterial dimensions in healthy cohorts, this study sets the stage for advancing automated vascular imaging, improving diagnostic accuracy, and reducing clinical workload. Our findings emphasize the transformative potential of this algorithm to standardize cerebrovascular imaging practices and enhance patient outcomes through technological innovation.

## Supplemental Material

sj-pdf-1-jcb-10.1177_0271678X251338972 - Supplemental material for Accurate and fully automated diameter measurements of Circle of Willis arteries on MRA imaging Supplemental material, sj-pdf-1-jcb-10.1177_0271678X251338972 for Accurate and fully automated diameter measurements of Circle of Willis arteries on MRA imaging by Julia Huck, Davy Vanderweyen, Tatjana Rundek, Mitchell SV Elkind, Jose Gutierrez, Maxime Descoteaux and Kevin Whittingstall in Journal of Cerebral Blood Flow & Metabolism

## Data Availability

The dataset used in this study is not publicly available due to ethical restrictions. The code used for analysis is available upon request.
