# Supplementary material for: Accurate and fully automated diameter measurements of Circle of Willis arteries on MRA imaging
Source: J Cereb Blood Flow Metab. 2025 May 5;45(9):1774–84. doi: 10.1177/0271678X251338972 (PMC12714687; doi:10.1177/0271678X251338972)
Supplement: sj-pdf-1-jcb-10.1177_0271678X251338972 - Supplemental material for Accurate and fully automated diameter measurements of Circle of Willis arteries on MRA imaging [file sj-pdf-1-jcb-10.1177_0271678X251338972.pdf]

Artery	*Slope*	*Intercept [mm]*	*R²*	*RSME [mm]*	*MAE [mm]*
ICA	0.66	1.03	0.36	0.43	0.34
ACA A1	0.71	0.58	0.19	0.24	0.32
MCA M1	0.38	1.69	0.21	0.24	0.19
PCom	0.64	0.50	0.28	0.37	0.28
PCA P1	0.53	0.87	0.20	0.31	0.23
BA	0.80	0.18	0.52	0.29	0.23

Table S1: Regression analysis results quantifying the relationship between eICAB diameters estimated at a 0.2083 mm isotropic voxel size and manual measurements. This table presents the slope, intercept, R², Root Mean Squared Error (RMSE), and Mean Absolute Error (MAE) for each artery, illustrating the predictive accuracy of the eICAB algorithm in estimating diameters.

		ICA	ACA A1	MCA M1	PCom	PCA P1	BA
All Participants	0.2	0.66mm ± 0.49mm (16.35% ± 12.49%)	-0.06mm ± 0.66mm (3.29% ± 24.84%)	0.04mm ± 0.35mm (1.98% ± 12.84%)	0.26mm ± 0.44mm (18.22% ± 26.52%)	0.30mm ± 0.39mm (15.98% ± 19.87%)	0.48mm ± 0.32mm (18.43% ± 12.91%)
	0.3125	0.78mm ± 0.47mm (19.23% ± 12.28%)	0.04mm ± 0.66mm (8.22% ± 26.17%)	0.2mm ± 0.36mm (7.93% ± 13.55%)	0.33mm ± 0.45mm (21.87% ± 27.53%)	0.4mm ± 0.39mm (20.7% ± 20.6%)	0.61mm ± 0.31mm (22.86% ± 13.0%)
	0.4	0.78mm ± 0.48mm (19.17% ± 12.48%)	0.12mm ± 0.66mm (12.54% ± 27.09%)	0.21mm ± 0.35mm (8.26% ± 13.39%)	0.43mm ± 0.44mm (27.75% ± 28.14%)	0.47mm ± 0.39mm (23.73% ± 21.26%)	0.61mm ± 0.32mm 23.16% ± 13.5%)
	0.5	0.88mm ± 0.46mm (21.59% ± 12.43%)	0.25mm ± 0.67mm (18.81% ± 28.63%)	0.34mm ± 0.36mm (13.28%± 14.12%)	0.52mm ± 0.45mm (32.9% ± 29.54%)	0.62mm ± 0.4mm (30.82%± 22.33%)	0.73mm ± 0.32mm (27.43% ± 14.13%)
	0.625	1.15mm ± 0.44mm (27.81% ± 12.44%)	0.39mm ± 0.66mm (25.71% ± 30.23%)	0.54mm ± 0.38mm (20.62% ± 15.0%)	0.72mm ± 0.44mm (44.2% ± 31.74%)	0.72mm ± 0.4mm (35.33% ± 23.03%)	0.92mm ± 0.31mm (34.43% ± 14.53%)
Participants with fetal PCA	0.2	0.56mm ± 0.56mm (13.75% ± 13.24%)	-0.08mm ± 0.71mm (3.30% ± 25.82%)	0.07mm ± 0.34mm (3.06% ± 12.79%)	0.23mm ± 0.51mm (15.15% ± 26.19%)	0.28mm ± 0.35mm (15.28% ± 20.88%)	0.47mm± 0.32mm (20.52% ± 15.03%)
	0.3125	0.67mm ± 0.53mm (16.3% ± 12.94%)	0.02mm ± 0.71mm (7.96% ± 26.82%)	0.22mm± 0.33mm (8.83% ± 12.91%)	0.3mm ± 0.55mm (18.74% ± 28.68%)	0.35mm ± 0.35mm (18.55% ± 20.67%)	0.59mm ± 0.33mm (25.58% ± 15.61%)
	0.4	0.69mm ± 0.55mm (16.72% ± 13.19%)	0.11mm ± 0.71mm (12.86% ± 28.12%)	0.23mm ± 0.34mm (9.1% ± 13.05%)	0.39mm ± 0.52mm (23.32% ± 28.87%)	0.43mm ± 0.32mm (22.53% ± 19.96%)	0.61mm ± 0.33mm (26.6% ± 16.05%)

	0.5	0.78mm ± 0.53mm (18.75% ± 13.03%)	0.24mm ± 0.72mm (19.07% ± 30.01%)	0.36mm± 0.35mm (13.92% ± 14.03%)	0.51mm ± 0.53mm (28.92% ± 29.21%)	0.58mm ± 0.35mm (29.53% ± 22.31%)	0.72mm ± 0.34mm (31.37% ± 16.87%)
	0.625	1.06mm ± 0.5mm (25.01% ± 13.04%)	0.37mm ± 0.71mm (25.79% ± 31.53%)	0.57mm± 0.36mm (21.52% ± 14.89%)	0.69mm ± 0.51mm (38.41% ± 30.6%)	0.68mm± 0.35mm (34.22% ± 22.95%)	0.91mm ± 0.33mm (39.3% ± 16.61%)

Table S2: Comparison of arterial diameters and percentage change in diameter for all participants and the fetal PCA subgroup. Mean ± standard deviation values are reported for each artery, along with the relative error (%) of the over/underestimation for the ICA, ACA A1, MCA M1, PCom, PCA P1, and BA.

	Left	Right
ICA	0.71mm ± 0.46mm (17.39% ± 12.04%)	0.62mm ± 0.52mm (15.31% ± 12.04%)
ACA A1	-0.12mm ± 0.69mm (1.05% ± 25.18%)	-0.00mm ± 0.61mm (5.60% ± 24.27%)
MCA M1	0.00mm ± 0.32mm (0.57% ± 12.10%)	0.07mm ± 0.37mm (3.39% ± 13.39%)
PCom	0.24mm ± 0.41mm (17.18% ± 25.38%)	0.27mm ± 0.47mm (19.04% ± 27.37%)
PCA P1	0.31mm ± 0.39mm (16.74% ± 20.74%)	0.29mm ± 0.40mm (15.14% ± 18.83%)
BA	0.48mm ± 0.32mm (18.43% ± 12.91%)	

Table S3: Comparison of arterial diameters and percentage changes in diameter between the left and right hemispheres across all participants
